# Association of leukocyte elastase in semen and seminal plasma with sperm parameters and pregnancy outcomes in male fertility

**DOI:** 10.1530/EC-24-0571

**Published:** 2025-01-27

**Authors:** Mausumi Das, Maha Gumssani, Julia Mullaney, Ralf Henkel, Suks Minhas, Marie Claire Aquilina, Channa N Jayasena

**Affiliations:** ^1^ Department of Metabolism, Digestion and Reproduction, Imperial College London, London, UK; ^2^ Department of Urology, Imperial College Healthcare, London, UK; ^3^ NIHR, Imperial Biomedical Research Centre, London, UK

**Keywords:** leukocyte elastase, sperm quality, pregnancy outcomes, infertility

## Abstract

**Background:**

Semen analysis is the standard test for evaluating male fertility. However, it may not address all aspects of male infertility. This review explores the role of leukocyte elastase (LE) as a possible biomarker for male fertility by evaluating 28 corresponding studies.

**Objectives:**

We aimed to explore how LE levels in semen relate to sperm quality and pregnancy outcomes.

**Methods:**

This systematic review followed PRISMA guidelines and included studies from PubMed, Medline, Embase and Scopus (March 22–25, 2024) using the keywords ‘elastase’, ‘sperm’ and ‘semen’. Out of 897 identified articles, 334 were screened, leading to 90 full-text reviews. We included 28 studies reporting sperm parameters linked to LE and excluded non-English articles, reviews and animal studies. Data collected included study details, methods, population, LE levels, sperm characteristics and pregnancy outcomes. A narrative synthesis was used because of differing study designs. Quality assessment, using the National Heart, Lung, and Blood Institute tool, rated 21 studies medium quality, 6 high and 1 low.

**Results:**

Only a limited number of studies reported a correlation between LE levels and sperm parameters, with no significant link to sperm concentration. Overall, we did not identify a strong association between LE levels and pregnancy or fertilization rates.

**Conclusions:**

Although LE serves as a marker for seminal leukocyte concentration, its link to sperm quality and fertility outcomes remains weak and inconsistent. Based on current evidence, LE does not appear to be a reliable diagnostic marker for male infertility. Future studies should focus on standardizing LE measurement techniques and exploring its interaction with other semen parameters to clarify its role in male fertility.

## Introduction

Infertility is a global health problem (1) and estimated to affect one in every seven couples (2) with approximately 40% of the cases solely attributable to a male factor (3). Male genital tract inflammation may significantly contribute to male infertility but is challenging to diagnose because of its frequent asymptomatic nature (4). Because genital tract infections can progress asymptomatically for years, they are often only incidentally detected through semen analysis when patients seek help at andrology units because of infertility. One of the signs of infection could be an elevated white blood cell count in the semen, measured by the Endtz test (5), where anything above 1 × 10^6^/mL indicates leukocytospermia (6). Semen cultures can also help identify bacterial infections (7). However, there is still a debate about whether leukocytospermia directly causes infertility or always requires treatment. The 2024 European Association of Urology (EAU) guidelines recommend distinguishing between infectious and noninfectious causes, prescribing antibiotics only for confirmed infections and using anti-inflammatory treatments for noninfectious inflammation. Routine screening for leukocytospermia is not necessary unless infection or inflammation is suspected (8). In asymptomatic men without infection, treatment is not needed unless issues such as oxidative stress are present (8). According to Boeri (2020), 20% of male partners of infertile couples expressed asymptomatic genital tract inflammation (9). Accordingly, an elevated number of leukocytes within the semen may indicate a male genitourinary infection with the potential to detrimentally impact fertility of the affected couple. However, using the leukocyte count as a risk assessment method for impaired fertility potential can pose significant challenges. Alternative methods, such as measuring leukocyte elastase (LE) levels in the seminal plasma, have shown that elevated LE (≥250–290 μg/L) is associated with genital tract infections, a pattern often observed in infertile men (10, 11, 12, 13, 14, 15). Advances in enzyme-linked immunosorbent assays have improved the differentiation between inflammatory and noninflammatory conditions in the seminal plasma (10).

Leukocytes primarily produce reactive oxygen species (ROS), which can be detrimental to sperm DNA and sperm cellular integrity (16, 17, 18). Consequently, higher levels of leukocytes may impair sperm quality (19, 20, 21, 22). However, studies have not confirmed this finding (10, 15, 23). Furthermore, there are a limited number of studies exploring the correlation between LE levels and sperm characteristics or pregnancy outcomes. Given this inconsistency and the limited number of studies, the current literature lacks clarity on the relationship between LE levels and fertility outcomes. To address this gap, we conducted a systematic review to investigate the potential link between LE levels in the seminal plasma and sperm parameters according to WHO criteria (6). We also investigated the evidence associating LE levels in the seminal plasma with pregnancy and embryological outcomes during assisted conception. We hypothesize that LE is significantly associated with abnormalities in sperm parameters and effects on pregnancy and embryological development.

## Methodology

**Figure 1:**
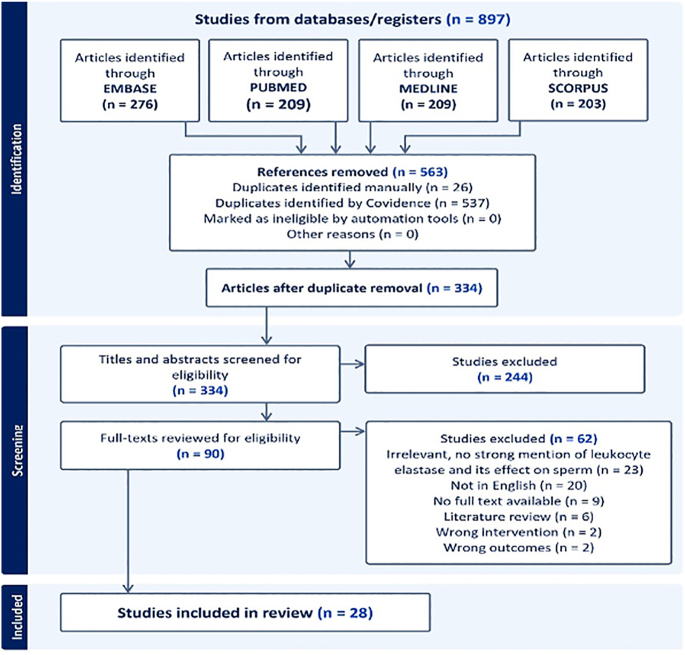
PRISMA flowchart. From an initial 897 articles, 563 were excluded during preliminary screening because of language barriers, irrelevant titles or lack of full-text access. After duplicates were removed, 334 articles were screened by title and abstract, leading to the exclusion of 244 that did not address sperm function, pregnancy outcomes or leukocyte elastase’s (LE’s) effect on male infertility. A total of 90 articles underwent full-text review, with 62 further excluded: 23 did not sufficiently focus on LE, 20 were not in English, 9 lacked full-text access, 6 were reviews and 4 had incorrect interventions or outcomes. Ultimately, 28 articles were included. Data from the 28 qualifying studies were gathered using a Covidence-based template.

This systematic review was conducted following the Preferred Reporting Items for Systematic Reviews and Meta-Analyses (PRISMA) guidelines. The database search was performed on PubMed, Medline, Embase and Scopus from March 22 to March 25, 2024. The systematic review aimed to investigate the correlation between LE levels in the semen and seminal plasma with sperm parameters and pregnancy outcomes. The search utilized the keywords ‘elastase’, AND ‘sperm’ AND ‘semen’. In our review, we have used Covidence (Covidence systematic review software, Veritas Health Innovation, Australia. Available at www.covidence.org) as it provides a standardized, consistent and reliable platform for conducting systematic reviews. The search results from PubMed, Medline, Embase and Scopus were imported into Covidence, and duplicates were removed both automatically by software and manually. Initially, the titles and abstracts of the studies were screened by three investigators (MD, MG and JM) before proceeding to a full-text review. Ninety full-text articles were independently reviewed by two investigators (MG and JM). Any discrepancies that emerged during the title screening and full-text review were discussed and resolved by all three authors. We included articles that reported on at least one semen/sperm parameter and focused on the relationship between LE and sperm parameters. Additionally, we considered articles that discussed LE in the context of infertility. Articles not written in English, as well as those without full text, literature reviews, conference abstracts and animal studies, were excluded from our review (Fig. 1: PRISMA flowchart).

In this systematic review, we included studies that explored the link between LE and sperm parameters or fertility outcomes, irrespective of whether they followed the most recent WHO guidelines (2021) for semen analysis. Most of the studies were published before the updated guidelines were released, so we did not specifically assess their adherence to the latest standards. However, we included them because they provided valuable data relevant to LE and its potential role in male infertility.

### Data synthesis

We used a narrative approach for data synthesis because of the differences in study designs and results, which made a meta-analysis unsuitable. The studies were grouped based on sperm parameters (e.g., concentration, motility, morphology, volume, DNA fragmentation and leukocyte concentration) and clinical outcomes (e.g., pregnancy rates and embryo development).

Data extraction was conducted by MG and JM through Covidence. We extracted relevant data from each study, including details such as authors, journal, study design, sample size, methods, population, intervention, LE levels, sperm characteristics and pregnancy results. The results were then summarized to highlight whether significant associations between LE and sperm parameters were found. Discrepancies between studies were examined, considering factors such as varying LE cut-off values. We also accounted for the quality of each study. Those with a higher risk of bias were interpreted cautiously to ensure the findings remain reliable. This narrative synthesis provides an overview of the relationship between LE levels and sperm parameters, as well as the potential implications for male infertility.

## Results

### Characteristics of studies

Out of the 897 articles initially identified, 563 were excluded during the preliminary screening because of factors such as language barriers, irrelevant titles or unavailability of full text. After removing duplicates, 334 articles were screened based on titles and abstracts for relevance. From these, 244 were excluded for not addressing sperm function or pregnancy outcomes in relation to LE or its broader impact on male infertility. This left 90 articles for a detailed full-text review, during which 62 studies were further excluded: 23 did not sufficiently discuss LE and its effects on sperm, 20 were not in English, 9 had no full text available, 6 were literature reviews, 2 involved incorrect interventions and 2 had incorrect outcomes. Consequently, 28 articles were suitable for inclusion in this review, as illustrated in the PRISMA flow chart (Fig. 1).

The systematic review encompassed 28 studies with a total of 13,716 participants, yielding an average sample size of 489.86 per study. All included studies were observational. Specifically, 14 were cross-sectional (51.9%), 10 were prospective cohort studies (37.0%), 2 were retrospective cohort studies (7.4%) and 1 was a cohort study (3.7%). Table 1 provides a breakdown of studies focusing on each parameter of interest: seminal LE concentration (*n* = 23) followed by total motility (*n* = 11), sperm concentration (*n* = 10), sperm morphology (*n* = 10), progressive motility (*n* = 10), semen volume (*n* = 9) and sperm count (*n* = 7). The sperm DNA fragmentation index (DFI), an important indicator of sperm quality, was the least studied parameter, with only four studies addressing it. Pregnancy-related outcomes, such as pregnancy and fertilization rates, were assessed in two studies. Miscarriage rates were evaluated in one study. Among the 28 studies, 16 specifically analyzed semen from men diagnosed with infertility, while 12 were conducted in andrological or fertility clinic settings. The risk of bias for each study was evaluated and is documented in (Supplementary Table 1 (see the section on Supplementary materials given at the end of the article)), with a comprehensive summary of the main findings provided in Table 2.

**Table 1:** Lists of the number of studies that investigated each parameter related to sperm, seminal parameters and pregnancy outcomes within the systematic review.

Sperm/semen parameter	No. of studies
Sperm concentration	10
Sperm count	7
Sperm morphology	10
Seminal volume	9
Progressive motility	10
Total motility	11
Elastase concentration	23
Sperm DFI	4
Pregnancy rates	2
Miscarriage rates	1
Fertilization rates	2

DFI, DNA fragmentation index.

**Table 2:** Studies investigating parameters related to sperm parameters and pregnancy outcomes.

Author	Journal	Study design	Findings
Eggert-Kruse 1996 (Germany)	*Fertility and Sterility*	Cross-sectional study	No significant correlation was found between antisperm antibodies and PMN granulocyte elastase levels in the seminal plasma nor with the results of semen cultures (52)
Eggert-Kruse 2009 (Germany)	*International Journal of Andrology*	Prospective cohort study	PMN elastase levels in both the serum and seminal plasma did not significantly affect semen quality. There was also no significant correlation between PMN elastase levels in the serum and seminal plasma nor any link to fertility outcomes. Additionally, high PMN elastase levels in male serum were not significantly associated with pregnancy rates or abortion rates (15)
Henkel 1998 (Germany)	*Andrologia*	Cross-sectional study	The data show a significant link between ROS production in viable sperm and the PMN elastase concentration. Multiple regression analysis, which focused on sperm motility, identified the count of round cells as the main factor influencing motility. The analysis determined a threshold of 49,489.9 counts of PMN elastase per 10^−7^ viable spermatozoa (53)
Henkel 2003 (Germany)	*Andrologia*	Cohort study	The study revealed that PMN elastase levels did not correlate with fertilization rates or pregnancy outcomes (13)
Henkel 2007 (Germany)	*Asian Journal of Andrology*	Retrospective cohort study	The results indicate a marked rise in PMN elastase levels, which serves as a marker of male genital tract inflammation in older men, suggesting age-related changes in local immune regulation. Additionally, the lack of association between PMN elastase and sperm motility suggests that a direct inhibitory effect of this enzyme can be ruled out (31)
Jochum 1986 (Germany)	*Andrologia*	Cross-sectional study	No correlation was found between PMN elastase levels and the percentage of morphologically intact spermatozoa, total sperm count or the pH and viscosity of the ejaculate (10)
Kopa 2005 (Hungary)	*Andrologia*	Prospective cohort study	Elastase concentrations were found to have a negative correlation with sperm vitality. Significant negative correlations were also observed with sperm motility, progressive motility and sperm morphology. Granulocyte elastase proves to be an effective marker for identifying silent genital tract inflammation (4)
Kratz 2020 (Poland)	*Journal of Physiology and Pharmacology*	Observational pilot study	Elevated average LE levels were noted in patients with oligozoospermia and teratozoospermia relative to those with normozoospermia. Despite these observed differences in mean and median LE values, they were not statistically significant enough to establish LE as a dependable biomarker (54)
Liu 2021 (China)	*Reproductive Biology and Endocrinology*	Retrospective cohort study	In comparison to the control group (no infection), the CT+, UU+ and CT/UU+ groups exhibited differences in semen volume, the proportion of sperm with normal morphology and measures of sperm motility, including both overall motility and progressive motility, as well as sperm vitality (27)
Liu 2022 (China)	*Journal of International Medical Research*	Prospective cohort study	Liu discovered that following a UU infection, changes in the PMN elastase concentration in the seminal plasma affect liquefaction time and viscosity, leading to reduced sperm motility (progressive motility) (26)
Maegawa 2001 (Japan)	*Archives of Andrology*	Cross-sectional study	Elastase levels showed no correlation with sperm motility (23)
Marconi 2009 (Germany)	*European Urology*	Prospective cohort study	Elastase levels exceeding 230 ng/mL did not reveal any notable variation in ASA levels compared with the control group. Additionally, no correlation was observed between chronic inflammation or infections in the MRT and the presence of ASA in the semen (55)
Micic 1989 (Yugoslavia, present day Serbia)	*International Journal of Andrology*	Cross-sectional study	Linear regression analysis revealed a positive correlation between LE levels in the seminal plasma and the leukocyte count in the ejaculate. The study, which assessed seminal plasma elastase levels in 84 infertile men using an immunoabsorbent assay, found significant improvements in the 58 men with elevated elastase levels after 6–12 weeks of antibiotic therapy. Specifically, reductions in elastase levels were strongly correlated with increases in the sperm count (*R* = −0.555; *P* < 0.01) and sperm motility (*R* = −0.477; *P* < 0.01). However, no significant change was observed in the percentage of sperm with normal morphology (*F* = 0.035; *P* > 0.05) (35)
Miska 1993 (Germany)	*Archives of Andrology*	Cross-sectional study	In a study of 79 adult men, those with PMN elastase levels between 201 and 700 pg/L showed higher percentages of global motility, normal motility and hypermotility compared with those with elastase levels between 0 and 200 pg/L. Specifically, the mean global motility was 47 ± 2% in the lower elastase group, while it increased to 57 ± 2% in the higher elastase group. Normal motility was 25 ± 2% in the lower elastase group and 30 ± 2% in the higher group, while hypermotility was 10 ± 1% and 17 ± 2% respectively. Conversely, hypomotility was lower in the higher elastase group, with 13 ± 1% in the 0–200 pg/L group and 10 ± 1% in the 201–700 pg/L group (25)
Moretti 2009 (Italy)	*International Journal of Andrology*	Cross-sectional study	Semen characteristics showed no correlation with PMN elastase levels. Although PMN elastase and ILs are typically associated with each other, no correlation was observed between these inflammatory mediators and semen parameters. Patients with idiopathic infertility exhibited altered semen quality despite having normal levels of inflammatory mediators (39)
Rajasekaran 1995 (USA)	*Fertility and Sterility*	Prospective cohort study	Infertile patients with leukocytospermia showed significantly elevated levels of granulocyte elastase and reactive oxygen species (ROS) in their semen (38)
Rajasekaran 1996 (USA)	*American Journal of Reproductive Immunology*	Prospective cohort study	A significant increase in granulocyte elastase activity (U/L) was observed in the seminal plasma of infertile patients with leukocytospermia, compared with normal fertile donors (*P* < 0.05) (37)
Reinhardt 1997 (Germany)	*Andrologia*	Cross-sectional study	A strong correlation was observed between elastase levels and several other inflammation markers. Although patients with elevated elastase levels showed impaired sperm motility and progressive motility, these changes were not statistically significant. The study demonstrates a notable correlation between elastase concentration in the seminal plasma and the number of white blood cells in semen, as well as with the seminal volume (12)
Ricci 2000 (Italy)	*Human Reproduction*	Cross-sectional study	No significant link was identified between semen parameters and the presence of leukocytospermia (34)
Shimoya 1993 (Japan)	*Fertility and Sterility*	Cross-sectional study	There was no statistically significant difference in PMN elastase levels between infertile men without leukospermia and fertile donors (36)
Tremellen 2010 (Australia)	*International Journal of Andrology*	Cross-sectional study	Seminal plasma elastase levels were significantly elevated in the infertile cohort. They found no correlation between seminal plasma elastase and any marker of sperm quality (30)
Wang 2022 (China)	*Medicine*	Cross-sectional study	At 600 ng/mL, there were significant differences in sperm morphology and sperm DFI between the two groups (*P* < 0.05). However, Spearman correlation analysis revealed no significant relationship between LE levels in semen and factors such as sperm kinetic parameters, sperm movement trajectory parameters, sperm morphological parameters and sperm DFI (*P* > 0.05). Using 600 ng/mL as a threshold for LE concentration in semen seems reasonable, as the correlation between LE levels and sperm quality does not appear significant (24)
Wolff 1990 (Germany)	*Fertility and Sterility*	Prospective cohort study	Patients with high granulocyte elastase levels in their semen (>1000 ng/mL; *n* = 26) showed a significant reduction in ejaculate volume, total sperm count and total motile sperm count. The study divided semen samples into two groups: one with normal to intermediate elastase levels (<1000 ng/mL; *n* = 150) and the other with elevated/pathologic elastase levels (>1000 ng/mL; *n* = 26). Those in the high granulocyte elastase group had notably lower ejaculate volume (*P* < 0.05), total sperm count (*P* < 0.05) and total motile sperm count (*P* < 0.05). However, there were no significant differences in sperm velocity or other semen characteristics (19)
Wolff 1991 (Germany)	*Fertility and Sterility*	Cross-sectional study	To assess the impact of inflammation, the group testing positive for *C. trachomatis* antibody was split into those with PMN elastase levels above 250 ng PMN-elastase/mL (*n* = 40) and those below 250 ng/mL (*n* = 33). In the higher inflammation group, semen parameters were significantly worse: ejaculate volume, 2.8 ± 0.2 mL compared to 3.3 ± 0.3 mL; progressive sperm motility was 18% ± 2% vs 24% ± 2%; and citric acid levels were 7.0 ± 0.7 mg vs 10.5 ± 1.4 mg per ejaculate (all parameters *P* < 0.05 by unpaired Student’s *t*-test). The group with *C. trachomatis* antibodies and levels elevated above 250 ng/mL showed marked decrease in both ejaculate volume and progressive sperm motility (56)
Zopfgen 2000 (Germany)	*Human Reproduction*	Cross-sectional study	Infertile patients had significantly higher PMN elastase levels, with concentrations at 102 μg/L compared to 48 μg/L (*n* = 234, *P* < 0.05). In the infertile group, the levels were even higher, at 144 μg/L vs 48 μg/L (*P* < 0.05). In eight of 40 infertile men with normozoospermia, PMN elastase concentrations were measured above 1000 μg/L. Despite this, there was no statistical relationship between the presence of microbiological findings and PMN elastase levels in the seminal plasma (57)
Zorn 2000 (Slovenia)	*Human Reproduction*	Prospective cohort study	A positive correlation was identified between the elastase concentration and patient age (*r* = 0.202, *P* = 0.0001). Similarly, elastase concentration showed a positive correlation with the leukocyte count. Conversely, there was a negative correlation between the elastase concentration and both semen volume and sperm single-stranded DNA. No significant relationship was found between the elastase concentration and the presence of antisperm antibodies nor was there any correlation between the elastase concentration and bacterial presence in the semen (11)
Zorn 2004 (Slovenia)	*International Journal of Andrology*	Prospective cohort study	They found a significant but weak association between s-EI levels and semen leukocytes (*r* = 0.49, *P* = 0.004) but not with traditional sperm characteristics. No correlation was observed between s-EI levels and fertilization rates. However, higher s-EI levels were linked to reduced blastocyst development (*P* = 0.03) and an increase in the number of arrested embryos (*P* = 0.04). Extending embryo culture to the blastocyst stage highlights the detrimental effects of clinically silent male genital tract inflammation on embryo developmental potential (46)
Zorn 2010 (Slovenia)	*Reproductive Biomedicine Online*	Prospective cohort study	High elastase concentration (290–1000 μg/L) were linked to a higher ROS index compared to low elastase levels (1.28 vs 1.01, *P* = 0.016). Slightly increases in leukocytes and elastase were associated with slightly poorer sperm quality, increased sperm necrosis, DNA damage, elevated intracellular ROS, and lower mitochondrial membrane potential (MMP). There were no linear correlations between seminal elastase levels and traditional sperm characteristics or sperm apoptotic markers. When men were grouped by elastase levels, very low elastase group (<100 μg/L, group 1, *n* = 137), low elastase group (100–289 μg/L, group 2, *n* = 50), high elastase group (290–999 μg/L, group 3, *n* = 48) and very high elastase group (>1000 μg/L, group 4, *n* = 16), no difference in classical sperm characteristics were observed between the groups. However, sperm ROS levels were significantly higher in group 3 compared to group 1 (58)

PMN, polymorphonuclear cells, a type of white blood cell involved in the inflammatory response; ROS, reactive oxygen species, which can damage sperm and affect fertility; ILs, interleukins; LE, leukocyte elastase, an enzyme linked to inflammation and sperm quality; ASA, antisperm antibodies, which can affect fertility by attacking sperm; MRT, male reproductive tract; DFI, DNA fragmentation index, a measure of sperm DNA integrity; CT+**,** subjects who tested positive for *Chlamydia trachomatis* infection; UU+**,** subjects who tested positive for *Ureaplasma urealyticum* infection; CT/UU+**,** subjects who tested positive for both *Chlamydia trachomatis* and *Ureaplasma urealyticum* infections.

### Quality assessment and risk of bias of studies

The quality and risk of bias for the 28 studies were evaluated using the Quality Assessment Tool for Observational Cohort and Cross-Sectional Studies from the National Heart, Lung, and Blood Institute (NHLBI). This tool provides a reproducible method for evaluating how accurately the study results reflect the impact of LE, while accounting for any potential design or execution flaws. Because the assessment process is somewhat subjective and lacks standardization, each study was reviewed based on the NHLBI’s questions, with responses marked as ‘yes’ or ‘no’. The overall quality rating of each study – low, medium or high – was determined by the number of ‘yes’ responses, with 0–5 indicating low quality, 6–10 indicating medium quality and 11–15 indicating high quality. The scoring system was adaptable, considering noncritical issues and the relevance of each question to the study. However, most studies were rated as medium quality, with 21 papers receiving this rating. Six papers were classified as high quality, while one paper was rated as low quality. Studies with low to medium quality ratings are considered to have a higher risk of bias, so their results and conclusions should be interpreted with caution. The complete quality assessment details are provided in Supplementary Table 1.

### Association of LE with seminal parameters and leukocyte concentration

In this systematic review, we looked at 14 studies that investigated how LE affects various sperm and seminal parameters. Among these, only Wang (2022) examined LE’s relationship with all the core sperm parameters (24). Ten studies focused on LE and sperm concentration, but none found a significant association (*P* > 0.05) (Table 5). However, among the studies that focused on the sperm count, only one study identified a significant difference (Table 6). Furthermore, out of nine studies examining LE and the seminal volume, five found a noteworthy negative correlation (*P* < 0.05), indicating that higher LE levels were linked to a lower seminal volume (Table 3). Among the studies focusing on sperm morphology, only two demonstrated a significant negative correlation with LE (Table 4). Similarly, of the ten studies that examined progressive motility, only three found a significant link (Table 7). In the case of total motility, 11 studies investigated the effect of LE, but just two revealed a significant relationship (Table 8). As for the sperm DFI, only one identified a negative association with LE (Table 9). On the other hand, among the ten studies that looked into LE levels and leukocyte concentration, three found a significant link, while four observed significant correlation at higher elastase levels (Table 10). Several studies sorted their samples based on different elastase cut-off values. They generally found that sperm parameters such as sperm count, volume and progressive motility were worse in samples with higher LE levels. However, even with increased LE, the sperm quality often remained within the normal range according to the WHO’s sixth-edition manual (6).

**Table 3:** Papers that investigated the correlation between LE and the seminal volume. Correlation analysis was conducted using Spearman’s correlation analysis. Significance levels were defined as follows: *P* < 0.05 (significant), *P* < 0.01 (highly significant) and *P* < 0.1 (tendency). Moretti (2009) divided the subjects into control and study groups and found significant differences in the study group between LE and the seminal volume. Note: the studies by Henkel (2007), Zorn (2000), Reinhardt (1997), Moretti (2009) and Wolf (1990) were the only ones to report a significant correlation with the seminal volume.

Studies	*p*	*r*	*n*	Statistical significance
Wang 2022	0.157	−0.066	460	Non-significant
Liu 2022				No correlation
Tremellen 2010		−0.075		Non-significant
Henkel 2007	<0.0001	−0.146	2113	Statistically significant
Zorn 2000	0.01	−0.146		Statistically significant
Ricci 2000		−0.2343	59	Non-significant
Reinhardt 1997	<0.05	0.32	63	Statistically significant
Moretti 2009	0.002		12	Statistically significant
Wolf 1990	<0.05		26	Statistically significant

*r*, correlation coefficient, indicating the strength and direction of the relationship between leukocyte elastase and the seminal volume; *p*, *P*-value representing the statistical significance of the correlation; *n*, sample size of the study; statistical significance levels: *P* < 0.05, statistically significant; *P* < 0.01, highly significant; *P* < 0.1, tendency.

**Table 4:** Papers that investigated the correlation between LE and sperm morphology. Zorn (2010) divided LE levels into three groups (low/medium/high) and found no significant differences between LE and sperm morphology. Note: Studies by Kopa (2005) and Wang (2022) were the only studies to report a significant correlation with sperm morphology at 250 ng/mL and 600 ng/mL respectively.

Studies	*p*	*r*	*n*	Statistical significance
Wang 2022	0.144	−0.068	460	Statistically significant*
Tremellen 2010		0.052		Non-significant
Kopa 2005	<0.05	−0.117		Statistically significant
Zorn 2000				No correlation
Ricci 2000		0.0673	59	Non-significant
Zopfgen 2000		0.02		Non-significant
Jochum 1986				Non-significant
Eggert-Kruse 2009				Non-significant
Reinhardt 1997				No correlation
Zorn 2010 (low/medium/high)				Non-significant

*p*, *P* value representing the statistical significance of the correlation; *r*, correlation coefficient, indicating the strength and direction of the relationship between leukocyte elastase (LE) and sperm morphology; *n*, sample size of the study; statistical significance levels: *P* < 0.05, statistically significant; no correlation, no significant relationship found in the study.

* Significant differences in sperm morphology between the two groups using an LE threshold of 600 ng/mL (*P* < 0.05). However, no significant differences were observed when comparing three groups with LE boundaries set at 250 and 1000 ng/mL.

**Table 5:** Papers investigating the correlation between LE and sperm concentration. Zorn (2010) categorized LE levels into three groups: low (*n* = 50), medium (*n* = 48) and high (*n* = 16). The study found no statistically significant differences in sperm progressive motility among these groups. Moretti (2009) divided subjects into control and non-control groups and found no significant differences in the sperm concentration between them.

Studies	*p*	*r*	*n*	Statistical significance
Wang 2022	0.648	0.021	460	Non-significant
Liu 2022				Non-significant
Tremellen 2010		0.079		Non-significant
Ricci 2000		0.04665	59	Non-significant
Moretti 2009	0.52		12	Non-significant
Wolf 1990	0.100		26	Non-significant
Eggert-Kruse 2009				Non-significant
Reinhardt 1997			63	Non-significant
Kratz 2020				Non-significant*
Zorn 2010 (low/medium/high)				Non-significant

Sperm concentration, the number of sperm per milliliter of ejaculate; *p*, *P*-value representing the statistical significance of the correlation; *r*, correlation coefficient, indicating the strength and direction of the relationship between leukocyte elastase (LE) and sperm concentration; *n*, sample size of the study; statistical significance levels: *P* < 0.05, statistically significant.

* LE was not significant in oligozoospermic patients.

**Table 6:** Studies Investigating the correlation between LE and the total sperm count. The study by Wolf (1990) was the only study to find a significant correlation with the total sperm count in the high elastase group >1000 ng/mL.

Studies	*p*	*r*	*n*	Statistical significance
Eggert-Kruse 2009				No correlation
Kopa 2005				No correlation
Zorn 2000				No correlation
Zopfgen 2000		0.07		Non-significant
Wolf 1990	<0.05		26	Statistically significant
Reinhardt 1997	>0.05		63	Non-significant
Jochum 1986				Non-significant

Total sperm count, the overall number of sperm; *p*, *P*-value representing the statistical significance of the correlation; *r*, correlation coefficient, indicating the strength and direction of the relationship between leukocyte elastase and the total sperm count; *n*, sample size of the study; statistical significance levels: *P* < 0.05, statistically significant; no correlation, no significant relationship found in the study.

**Table 7:** Studies investigating the correlation between LE and sperm progressive motility. Zorn (2010) divided LE levels into low (*n* = 50), medium (*n* = 48) and high (*n* = 16) groups and found no significant differences in sperm progressive motility. Notably, Liu (2022), Kopa (2005) and Miska (1993) were the only studies to report a significant correlation with sperm progressive motility.

Studies	*p*	*r*	*n*	Statistical significance
Wang 2022	0.784	0.013	460	Non-significant
Liu 2022	<0.001	−0.89		Statistically significant
Eggert-Kruse 2009				No correlation
Henkel 2007	0.859	0.004	1767	Non-significant
Kopa 2005	<0.05	−0.112		Statistically significant
Zopfgen 2000		0.04		No correlation
Reinhardt 1997	>0.05			Non-significant
Zorn 2010				Non-significant
Wolff 1990			26	Non-significant
Miska 1993			28	Statistically significant*

*p*, *P*-value representing the statistical significance of the correlation; *r*, correlation coefficient, indicating the strength and direction of the relationship between leukocyte elastase and sperm progressive motility; *n*, sample size of the study; statistical significance levels: *P* < 0.05, statistically significant; no correlation, no significant relationship found in the study.

* Sperm hypermotility was statistically significant in 28 men with PMN elastase levels between 201 and 700 pg/L.

**Table 8:** Studies investigating the correlation between LE and sperm total motility. Moretti (2009) categorized subjects into control (*n* = 11) and non-control groups (*n* = 12) but found no significant differences in sperm total motility. Notably, Kopa (2005) and Miska 1993 were the only studies to find a significant correlation with sperm total motility.

Studies	*p*	*R*	*n*	Statistical significance
Wang 2022	0.905	0.006	460	Non-significant
Tremellen 2010		−0.014		Non-significant
Eggert-Kruse 2009				No correlation
Henkel 2007	0.2191	0.029	1773	Non-significant
Kopa 2005	<0.05	−0.112		Statistically significant
Maegawa 2001	0.42	0.116	116	Non-significant
Ricci 2000		0.0738	59	Non-significant
Reinhardt 1997	>0.05		63	Non-significant
Moretti 2009	0.54		12	Non-significant
Wolff 1990			26	No significant
Miska 1993			28	Statistically significant*

*p*, *P*-value representing the statistical significance of the correlation; *r*, correlation coefficient, indicating the strength and direction of the relationship between leukocyte elastase and sperm total motility; *n*, sample size of the study; statistical significance levels: *P* < 0.05, statistically significant; no correlation, no significant relationship found in the study.

* Sperm global motility was statistically significantly higher in men having PMN elastase levels between 201 and 700 pg/L.

**Table 9:** Papers that investigated the correlation between LE and the sperm DFI. Note: The study by Kopa (2005) was the only study to report a significant correlation with the sperm DFI.

Studies	*p*	*r*	*n*	Statistical significance
Wang 2022	0.674	0.02		Non-significant
Tremellen 2010		0.109		Non-significant
Kopa 2005	<0.05	−0.643		Statistically significant

*p*, *P*-value representing the statistical significance of the correlation; *r*, correlation coefficient, indicating the strength and direction of the relationship between leukocyte elastase and the sperm DNA fragmentation index (DFI); *n*, sample size of the study; statistical significance levels: *P* < 0.05, statistically significant.

**Table 10:** Studies on the correlation between leukocyte elastase (LE) and leukocyte concentration. A) studies comparing LE with leukocyte concentration. The studies by Zorn (2000), Ricci (2000) and Micic (1989) were the only ones to show a significant correlation with leukocyte concentration. B) Studies dividing LE into three groups (low/medium/high) and comparing with leukocyte concentration. Reinhardt (1997), Shimoya (1993), Rajasekaran (1995) and Rajasekaran (1996) observed significant differences associated with high elastase levels.

Studies	*p*	*r*	*n*	Statistical significance
Eggert-Kruse 2009				No correlation
Zorn 2000	<0.0001	0.33		Statistically significant
Ricci 2000	<0.001	0.542	59	Statistically significant
Micic 1989	<0.01	0.601		Statistically significant
Henkel 1998	0.378	0.1311	94	Non-significant

Studies	Zorn 2010	Shimoya 1993	Rajasekaran 1996 (study group)	Reinhardt 1997	Rajasekaran 1995
Low elastase			>0.05		
*n*	50	34	10	108	
Statistically significant	Non-significant	Non-significant	Non-significant		
Medium elastase					
*n*	48			134	
Statistically significant	Non-significant				
High elastase		<0.0001	<0.05		<0.05
*n*	16	10	12	63	
Statistically significant	Non-significant	Statistically significant	Statistically significant	Statistically significant	Statistically significant

*p*, *P*-value representing the statistical significance of the correlation; *r*, correlation coefficient, indicating the strength and direction of the relationship between LE and leukocyte concentration; *n*, sample size of the study; statistical significance levels: *P* < 0.05, statistically significant; *P* > 0.05, non-significant.

### Association of LE with clinical outcomes

Two studies examined the effect of LE on pregnancy outcomes, while one study explored its effect on miscarriage rates. None of these studies found a significant link between LE levels and pregnancy, fertilization or miscarriage rates.

## Discussion

WHO semen analysis remains the gold-standard test for male fertility potential but requires technical expertise to conduct rigorously and is unlikely to explain all forms of male infertility. Evaluating potentially novel markers of male fertility may therefore support the clinical evaluation of couples with infertility. Our analysis of 28 eligible studies has identified that seminal LE levels were correlated with semen volume, leukocyte concentration, total sperm count, motility, morphology and DNA fragmentation but not with sperm concentration.

We identified studies suggesting a possible link between LE and some parameters of sperm quality. Kopa (2005) and Miska (1993) reported significant correlations between LE levels and total sperm motility (4, 25). Similarly, only three studies reported a significant correlation between LE levels and progressive sperm motility (4, 25, 26). This detrimental effect of LE on sperm is likely because of an increase in ROS. Excessive ROS leads to lipid peroxidation, which in turn increases the permeability of spermatozoa membranes. Consequently, the sperm membranes lose their structural integrity and forward motility (27). Several studies have explored the link between LE and the sperm DFI. Among these, only one demonstrated a significantly negative effect of LE on sperm DNA integrity (4). LE indirectly causes DNA damage by increasing the production of ROS, which has been previously associated with an elevated sperm DFI (27). This damage is primarily because of oxidative stress and repair failure of guanine bases (28). Kopa (2005) and Wang (2022) were the only studies to find a correlation between LE and a reduction in normal sperm morphology, with cut-off values of 250 and 600 ng/mL respectively (4, 24). ROS, as previously mentioned, may harm sperm morphology by disrupting the lipid membrane and compromising chromatin integrity, which in turn affects both the structure and functional capability of the sperm.

We observed variables in the relationships between LE levels and sperm characteristics, which may be attributed to several factors. One major factor is the protective role of leukocyte elastase inhibitors (LEIs). For instance, while LE may initially reduce sperm motility, Moriyama (1998) demonstrated that sperm motility improved after the addition of LEIs (29). These inhibitors, produced by the body during inflammation, neutralize the damaging effects of elastase, thus shielding sperm from potential harm (29). Additionally, variations in cut-off values for defining LE levels have led to inconsistent results. Some studies used thresholds of 250 to 1000 ng/mL, which contributed to confusion in diagnosing and interpreting the effect of LE on sperm quality (11, 30, 31). For example, Wang (2022) found no significant differences in semen quality when using a cut-off of 250 ng/m, but observed significant differences at 600 ng/mL according to European standards (24). Another consideration is that LE levels may rise only when leukocytes are activated (32), and some studies might have included partially activated leukocytes. Variability in individual responses to infections and inflammation complicates establishing a consistent link between LE levels and sperm quality, as some people may exhibit greater resistance to increases in LE (24). Finally, inflammation often occurs in the epididymis, prostate or seminal vesicles rather than in the testis, which is protected by a robust blood–testis barrier (19). This barrier helps maintain a stable environment within the testes, crucial for sperm production. Therefore, LE-derived ROS are only present in the sperm during ejaculation, which is not a sufficient duration to significantly affect sperm quality (33). Additionally, most studies focused on asymptomatic patients, which may have led to different outcomes compared with studies involving those with clinical symptoms of genital tract inflammation.

Our systematic review showed contradictory results regarding LE levels and leukocyte concentration. Among the ten studies analyzing LE levels and leukocyte concentration, three (11, 34, 35) reported a significant correlation. Additionally, studies by Shimoya (1993), Rajasekaran (1995, 1996) and Reinhardt (1997) observed significant correlations at higher elastase levels (12, 36, 37, 38). These inconsistencies might be because of the studies using different measurement techniques and thresholds. Zorn (2000) demonstrated a strong connection between elastase concentrations and inflammation using a threshold of 290 ng/mL (11). They found that relying only on leukocytospermia to detect inflammation missed certain cases (11). Therefore, LE may be a more accurate marker than leukocyte counts alone because it reflects the functional activity of neutrophils and their role in inflammation, which includes proteolytic activity and the production of ROS that can damage the sperm membrane and contribute to infertility. Furthermore, this marker could prove useful in cases of subclinical inflammation, where leukocytospermia might not be detected, wherein the patient may still present low leukocyte counts with elevated leukocyte activity. Identifying such hidden inflammation may aid physicians in starting antibiotics, ultimately improving sperm quality and fertility outcomes. In addition, our systematic review also found that there might be an association between LE levels and decreased seminal volume. Out of nine studies that looked into this, only five found a link between higher LE levels and reduced seminal volume (11, 12, 19, 31, 39). Moretti (2009) provided particularly significant results by comparing samples from men with infections to those without (39). They found that men with genitourinary infections had a significantly lower seminal volume compared with the control group (39). Although LE-induced prostate inflammation does not directly affect sperm production, the prostate is vital for semen production, contributing about 25% of the ejaculate through prostatic fluid secretion (40). Infections such as chronic bacterial prostatitis and Chlamydia trachomatis have been linked to impaired semen production because they cause inflammation in the prostate, which reduces its fluid output and lowers the ejaculate volume (40). Moreover, the notable drop in the ejaculate volume seen in some studies may also be related to reduced free testosterone levels, a factor often observed (41, 42). These factors together suggest that LE-related prostate inflammation could also lead to a reduced seminal volume.

On the other hand, among the 28 studies reviewed, only two examined the link between LE and outcomes related to pregnancy and embryogenesis (13, 15), and one study investigated its link to miscarriage rates (15). None of the studies found a correlation between LE levels and pregnancy outcomes. Sperm separation methods used prior to IVF effectively reduce sperm exposure to potentially damaging levels of leukocytes and ROS. As a result, significant effects on fertilization and pregnancy are likely only in cases with exceptionally high leukocyte levels (13). Fertilization outcomes are also negatively affected by sperm quality, especially when motility is low (43, 44) or morphology is abnormal (45). Interestingly, one study found that elevated LE inhibitors levels were associated with poorer blastocyst development rates and a higher incidence of arrested embryos when the LEI concentration was >250 μg/mL (46). However, it is crucial to remember that blastocyst formation is also influenced by factors such as the number and maturity of oocytes retrieved and the female’s age (47). Although there is a lack of studies specifically examining the effects of LE on pregnancy, we can infer potential effects of leukocytes on fertility. Sukcharoen (1995) found that high leukocyte levels in semen are associated with reduced fertilization rates during IVF (48). Furthermore, Aitken (1996) observed that removing leukocytes from sperm suspensions using magnetic beads coated with an anti-CD45 antibody significantly improved sperm–oocyte fusion rates (49). Conversely, some research studies suggest that leukocytes may aid fertility by eliminating abnormal sperm and producing hydrogen peroxide, which is crucial for sperm capacitation (50, 51). The conflicting findings regarding LE’s effect on sperm may be because of significant differences in study methodologies. Sample sizes varied widely, from as few as 12 to as many as 4265 patients. Various methods were used to measure LE levels, such as ELISA, polymorphonuclear elastase assays and microplate readers. Additionally, different studies set different cut-off values for LE, making it difficult to consistently interpret the effects of high vs low elastase levels on sperm. To better understand LE’s impact on sperm and semen parameters, it is essential to establish a standardized cut-off value for elastase, allowing for more reliable comparisons between studies.

In conclusion, while the WHO semen analysis remains the benchmark for assessing male fertility, it falls short in explaining all forms of infertility, underscoring the need to explore additional markers such as LE. Although leukocytospermia, indicated by elevated leukocyte counts above 1 × 10^6^/mL (24), is commonly associated with decreased sperm quality (24), the role of LE as a specific marker of inflammation requires more careful consideration. The Endtz test, used to identify leukocytospermia, only detects granulocytes, which limits its effectiveness in providing a complete assessment of white blood cell involvement in male fertility (7).

Our review of 28 studies highlights that although LE consistently serves as a marker for seminal leukocyte concentration, its relationship with key sperm parameters such as count, motility, morphology and DNA fragmentation remains weak and inconsistent, with limited clinical utility. Furthermore, no significant correlation was found between LE levels and fertility outcomes such as pregnancy or fertilization rates. These findings challenge the value of LE as a meaningful predictor of male fertility. The variability in findings can largely be attributed to significant differences in methodologies, sample sizes and LE measurement techniques across studies. Additionally, the presence of elastase inhibitors and the protective barriers within the reproductive system further complicate the interpretation of LE’s effects.

Based on the current evidence, LE does not appear to be a reliable diagnostic marker for male infertility. Although it may provide some insight into inflammation-related conditions, the observed correlations are often weak and influenced by methodological inconsistencies. Future research should prioritize developing standardized thresholds for LE levels and establishing its sensitivity and specificity when used alongside semen parameters. It is also important to explore how leukocyte activation and inflammation affect sperm quality in larger, well-designed studies. Furthermore, there is also increasing interest in the relationship between LE and seminal dysbiosis, which refers to the imbalance of microbial populations in the semen. This dysbiosis could potentially raise LE levels because of the associated inflammation. If a strong connection is established, LE might become an important biomarker for diagnosing both dysbiosis and infertility, highlighting its growing significance in reproductive health (40).

It is important to acknowledge the strengths and limitations of our study. To our knowledge, this is the first review to evaluate the influence of LE on sperm and seminal parameters, uncovering a notable gap in the literature concerning LE’s effects on fertilization and pregnancy outcomes. One of the main challenges was the absence of standardized LE cut-off values for male fertility, which made it difficult to compare data across studies. Additionally, differences in methodologies, inclusion criteria and sample sizes contributed to inconsistent findings. Even though all the studies reviewed focused on sperm quality in men with elevated LE levels, only about half actually used statistical tests to directly investigate the link between LE and poor sperm parameters. Furthermore, some of the studies included in this review were published before the 2021 WHO guidelines for semen analysis were introduced. As a result, they may not have adhered to the updated standards. Although we did not specifically assess compliance with these guidelines, we acknowledge that differences in study methodologies could have affected the consistency of the results. Nevertheless, these studies were selected for their relevance to the topic of LE and male infertility, and they offer valuable insights despite the methodological differences.

## Supplementary materials



## Declaration of interest

The authors declare that there is no conflict of interest that could be perceived as prejudicing the impartiality of the work.

## Funding

This work did not receive any specific grant from any funding agency in the public, commercial or not-for-profit sector.

## Author contribution statement

MD came up with the study design and methodology, conducted the literature search, assisted in the screening of titles and abstracts, participated in resolving discrepancies during the full-text review process, performed analysis and interpretation of data, assisted in analyzing the quality of included studies and critically reviewed and revised the manuscript. MG drafted the manuscript, conducted the literature search, screened manuscripts, performed data extraction, conducted full-text review of articles, analyzed the quality of the included studies, performed data synthesis and interpretation, reviewed the results and participated in resolving discrepancies during the full-text review process. JM conducted the literature search, screened manuscripts, carried out data extraction, conducted full-text review of articles and analyzed the quality of the included studies, performed data synthesis and participated in resolving discrepancies during the full-text review process. CJ, SM, RH and MA critically reviewed the manuscript and provided feedback. All authors were involved in the final approval of the article.
