# Supplementary material for: Association of leukocyte elastase in semen and seminal plasma with sperm parameters and pregnancy outcomes in male fertility
Source: Endocr Connect. 2025 Jan 27;14(2):e240571. doi: 10.1530/EC-24-0571 (PMC11799752; doi:10.1530/EC-24-0571)
Supplement: Supplementary file 1 [file supplementary_materials.pdf]

SUPPLEMENTARY TABLE 1. Quality Assessment of 28 studies included in the systematic review. Papers were determined by the number of 'Yes' responses, alongside the non-critical 'No' and 'NA' responses. Observational studies were automatically assigned a 'No,' which is not seen as a critical issue since these studies, being exploratory, do not typically include information on power or sample size. Similarly, cross-sectional studies received an automatic 'No' due to their weaker design compared to cohort studies, which should be considered when evaluating the potential for bias. Abbreviations used include CD for 'cannot determine,' NA for 'not applicable,' and NR for 'not reported.

Was the research question or objective in this paper clearly stated?	Was the study population clearly specifed and defined?	Was the participation rate of eligible persons at least 50%?	Were all the subjects selected or recruited from the same or similar	Were inclusion and exclusion criteria for being in the study prespecified?	Was a sample size justification, power description, or variance and effect	For the analyses in this paper, were the exposure(s) of interest measured	Was the timeframe sufficient so that one could reasonably expect to see an	For exposures that can vary in amount or level, did the study examine	Were the exposure measures (independent variables) clearly defined, valid,	Was the exposure(s) assessed more than once over time?	Were the outcome measures (dependent variables) clearly defined, valid,	Were the outcome assessors blinded to the exposure status of participants?	Was loss to follow-up after baseline 20% or less?	Were key potential confounding variables measured and adjusted	Quality rating

Eggert-Kruse 1996	Eggert-Kruse 2009	Henkel 1998	Henkel 2003	Henkel 2007	Jochum 1986	Kopa 2005
Yes	Yes	Yes	No	Yes	Yes	Yes
Yes	Yes	No	No	Yes	Yes	Yes
Yes	Yes	Yes	Yes	Yes	Yes	Yes
Yes	Yes	Yes	Yes	Yes	Yes	Yes
Yes	Yes	No	No	No	No	No
No	No	No	No	No	No	No
No	Yes	No	Yes	Yes	No	Yes
Yes	Yes	Yes	Yes	Yes	Yes	Yes
NA	NA	No	NA	NA	NA	NA
Yes	Yes	Yes	No	Yes	Yes	Yes
NA	NA	NA	NA	NA	NA	NA
Yes	Yes	Yes	Yes	Yes	Yes	Yes
No	No	No	No	No	No	No
Yes	Yes	Yes	No	Yes	Yes	Yes
Yes	Yes	NR	NR	Yes	Yes	Yes
**Medium**	**Medium**	**Medium**	**Low**	**Medium**	**Medium**	**Medium**

Kratz 2020	Liu 2021	Liu 2022	Maegawa 2001	Marconi 2009	Micic 1989	Miska 1993	Moretti 2009
Yes	Yes	Yes	Yes	Yes	Yes	Yes	Yes
Yes	Yes	Yes	No	Yes	Yes	Yes	No
Yes	Yes	Yes	Yes	Yes	Yes	Yes	Yes
Yes	Yes	Yes	Yes	Yes	Yes	Yes	Yes
Yes	Yes	Yes	No	No	No	No	Yes
No	No	No	No	No	No	No	No
Yes	Yes	Yes	No	Yes	No	No	No
Yes	Yes	Yes	Yes	Yes	Yes	Yes	Yes
NA	NA	NA	No	NA	NA	No	NA
Yes	Yes	Yes	Yes	Yes	Yes	Yes	Yes
NA	NA	NA	NA	NA	NA	NA	NA
Yes	Yes	Yes	Yes	Yes	Yes	Yes	No
No	No	No	No	No	No	No	No
Yes	Yes	Yes	Yes	Yes	Yes	Yes	Yes
Yes	Yes	Yes	NR	Yes	Yes	Yes	NR
**High**	**High**	**High**	**Medium**	**Medium**	**Medium**	**Medium**	**Medium**

**Rajasekaran 1995**	**Rajasekaran 1996**	**Reinhardt 1997**	**Ricci 2000**	**Shimoya 1993**	**Tremellen 2010**
Yes	Yes	Yes	Yes	Yes	Yes
No	No	Yes	Yes	No	Yes
Yes	Yes	Yes	Yes	Yes	Yes
Yes	Yes	Yes	Yes	Yes	Yes
No	No	Yes	No	Yes	Yes
No	No	No	No	No	No
Yes	Yes	No	No	No	No
Yes	Yes	Yes	Yes	Yes	Yes
NA	No	NA	NA	NA	NA
Yes	Yes	Yes	Yes	Yes	Yes
NA	NA	NA	NA	NA	NA
Yes	Yes	Yes	Yes	Yes	Yes
No	No	No	No	No	No
Yes	Yes	Yes	Yes	Yes	Yes
NR	NR	Yes	Yes	NR	Yes
**Medium**	**Medium**	**Medium**	**Medium**	**Medium**	**Medium**

**Wang 2022**	**Wolff 1990**	**Wolff 1991**	**Zopfgen 2000**	**Zorn 2000**	**Zorn 2004**	**Zorn 2010**
Yes	Yes	Yes	Yes	Yes	Yes	Yes
Yes	No	No	Yes	Yes	Yes	Yes
Yes	Yes	Yes	Yes	Yes	Yes	Yes
Yes	Yes	Yes	Yes	Yes	Yes	Yes
No	No	No	No	Yes	Yes	Yes
No	No	No	No	No	No	No
No	Yes	No	No	Yes	Yes	Yes
Yes	Yes	Yes	Yes	Yes	Yes	Yes
No	No	No	NA	NA	NA	Yes
Yes	Yes	Yes	Yes	Yes	Yes	Yes
NA	NA	NA	NA	NA	NA	NA
Yes	Yes	Yes	Yes	Yes	Yes	Yes
No	No	No	No	No	No	No
Yes	Yes	Yes	Yes	Yes	Yes	Yes
Yes	NR	NR	Yes	Yes	Yes	Yes
**Medium**	**Medium**	**Medium**	**Medium**	**High**	**High**	**High**
